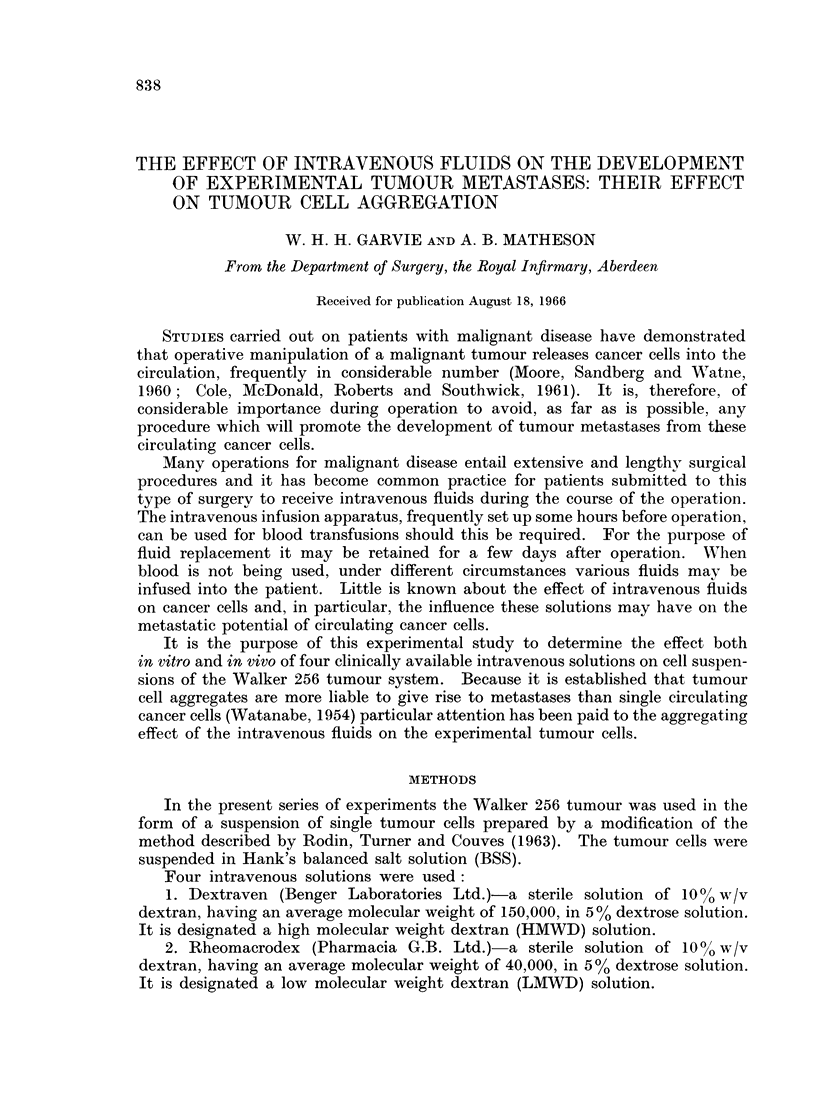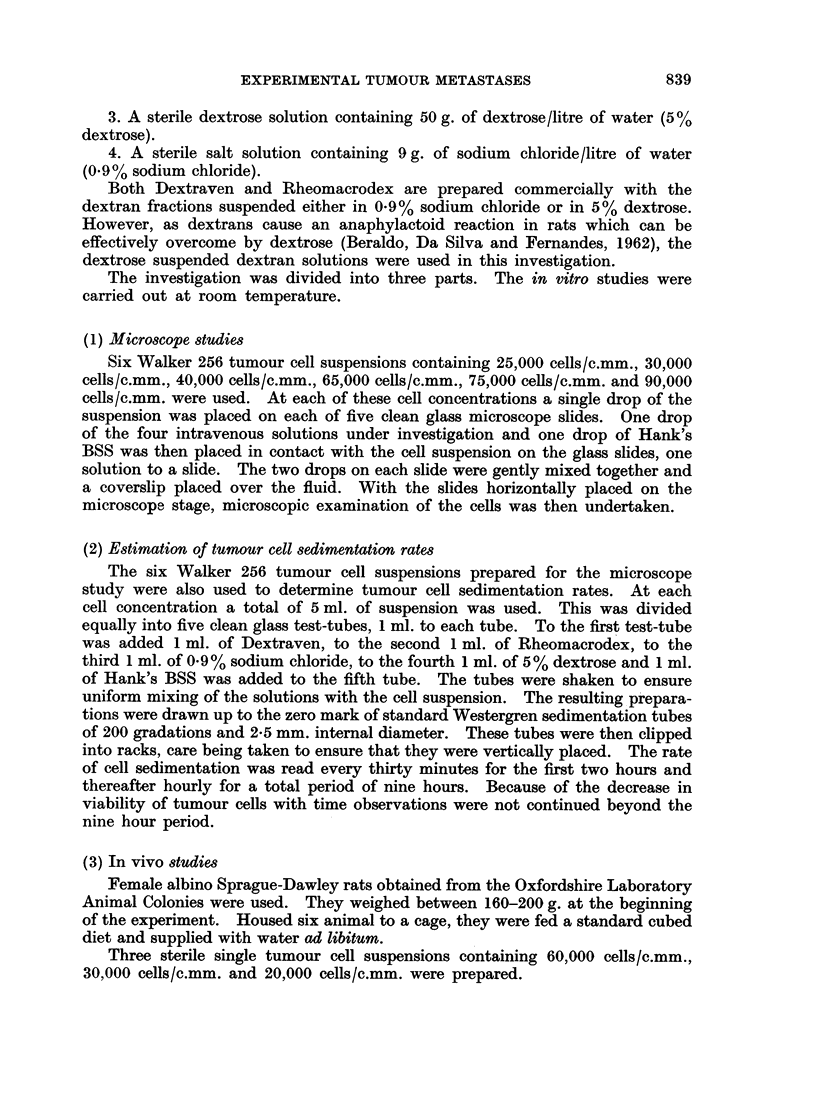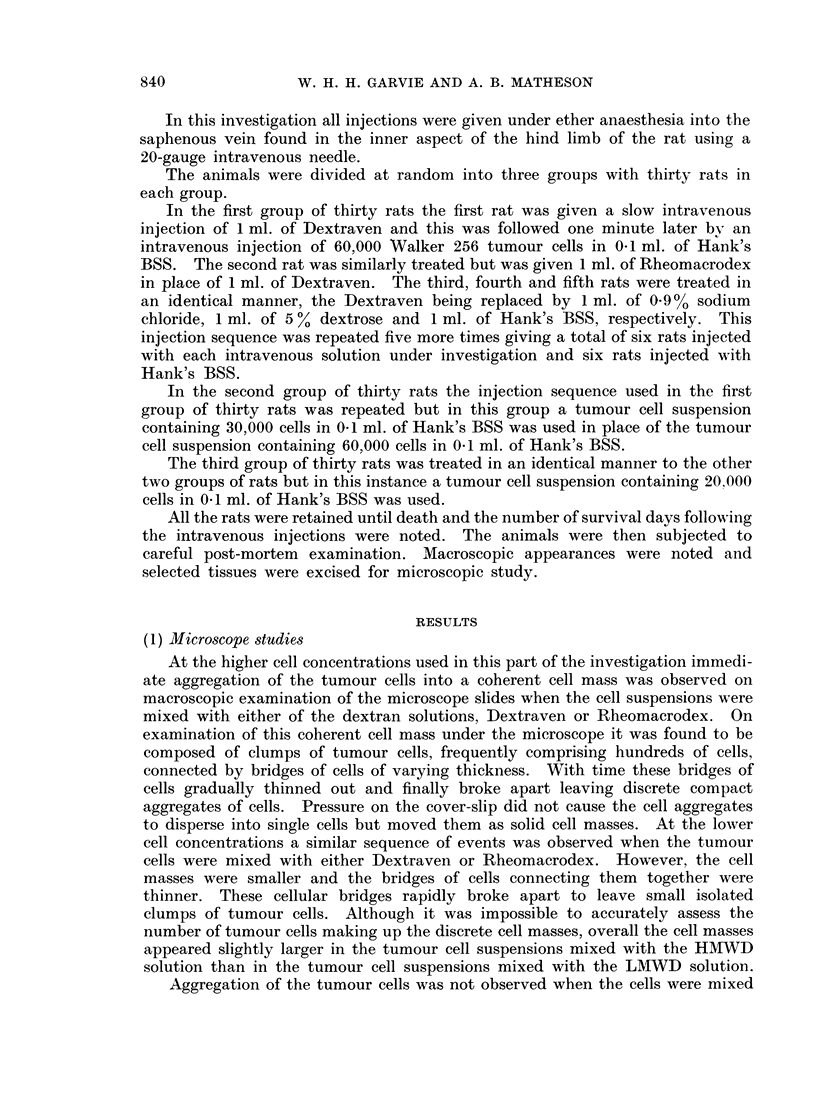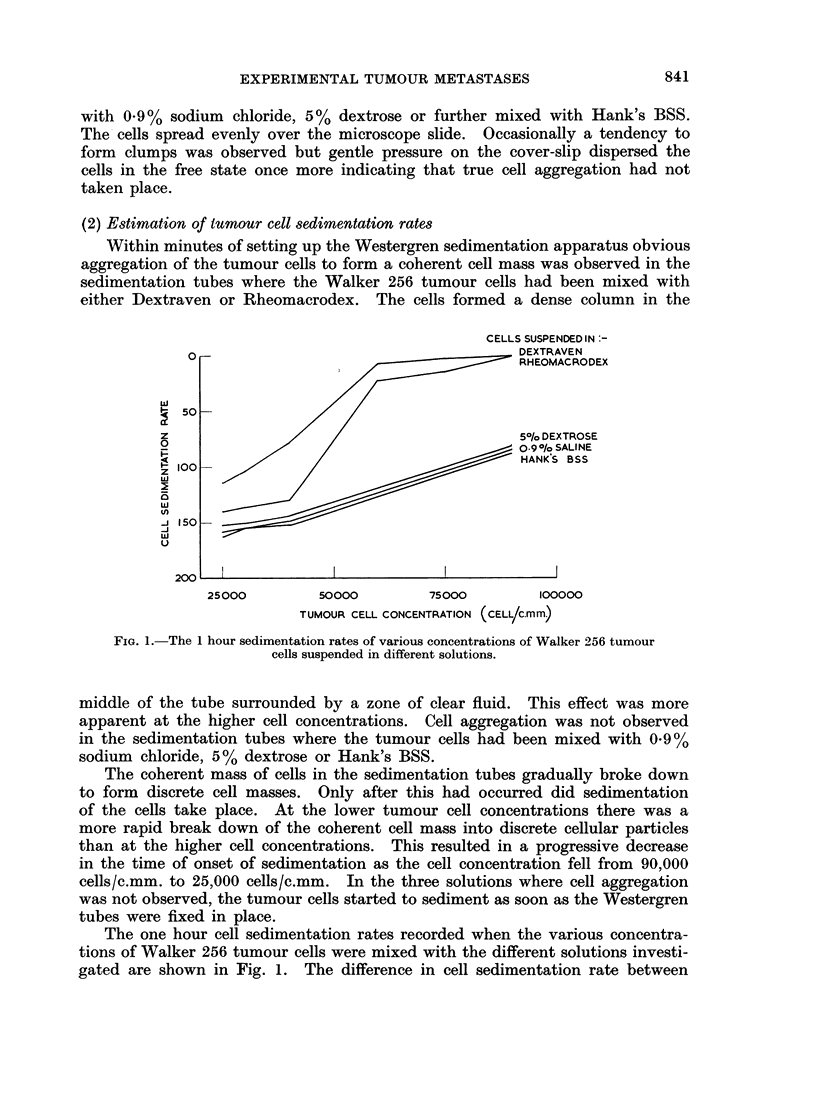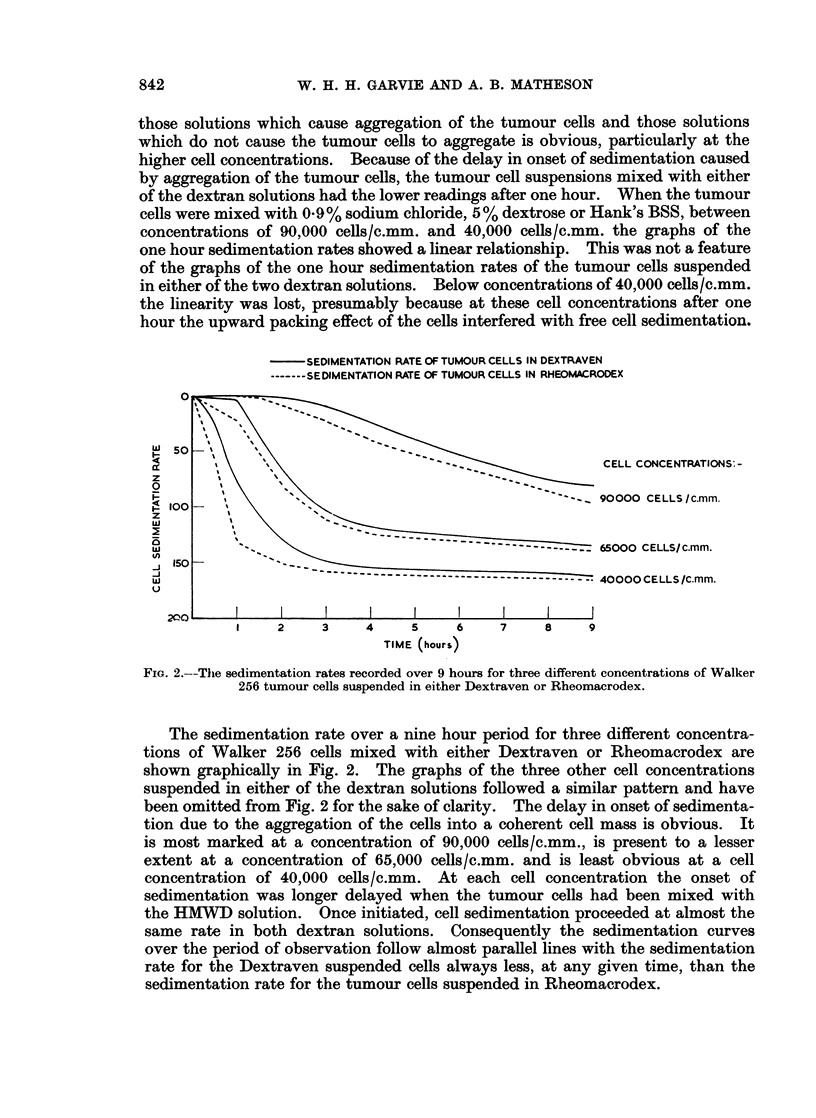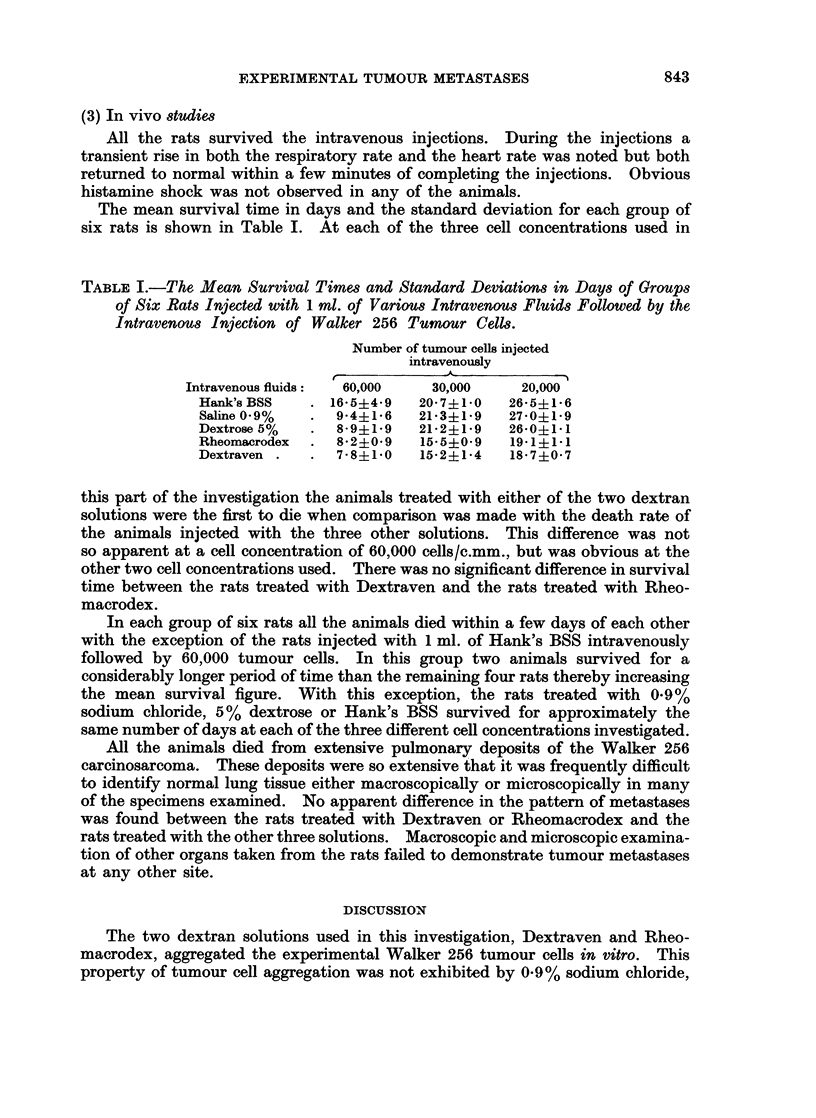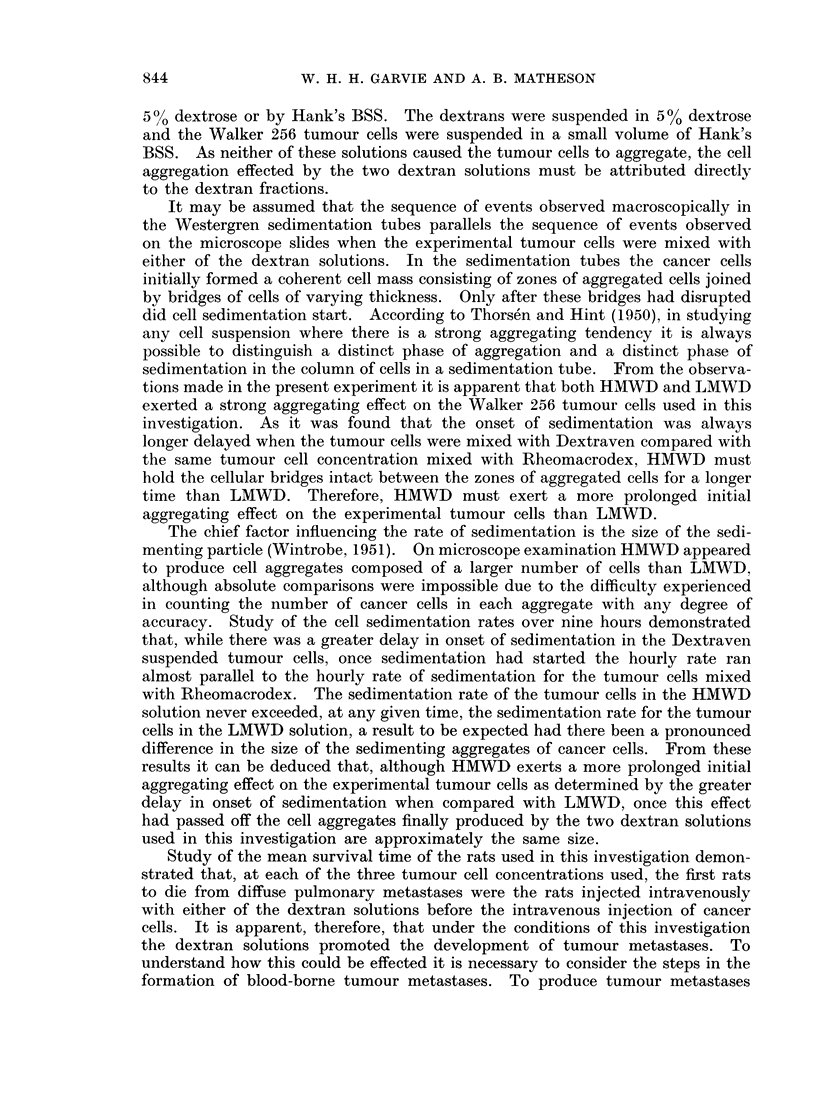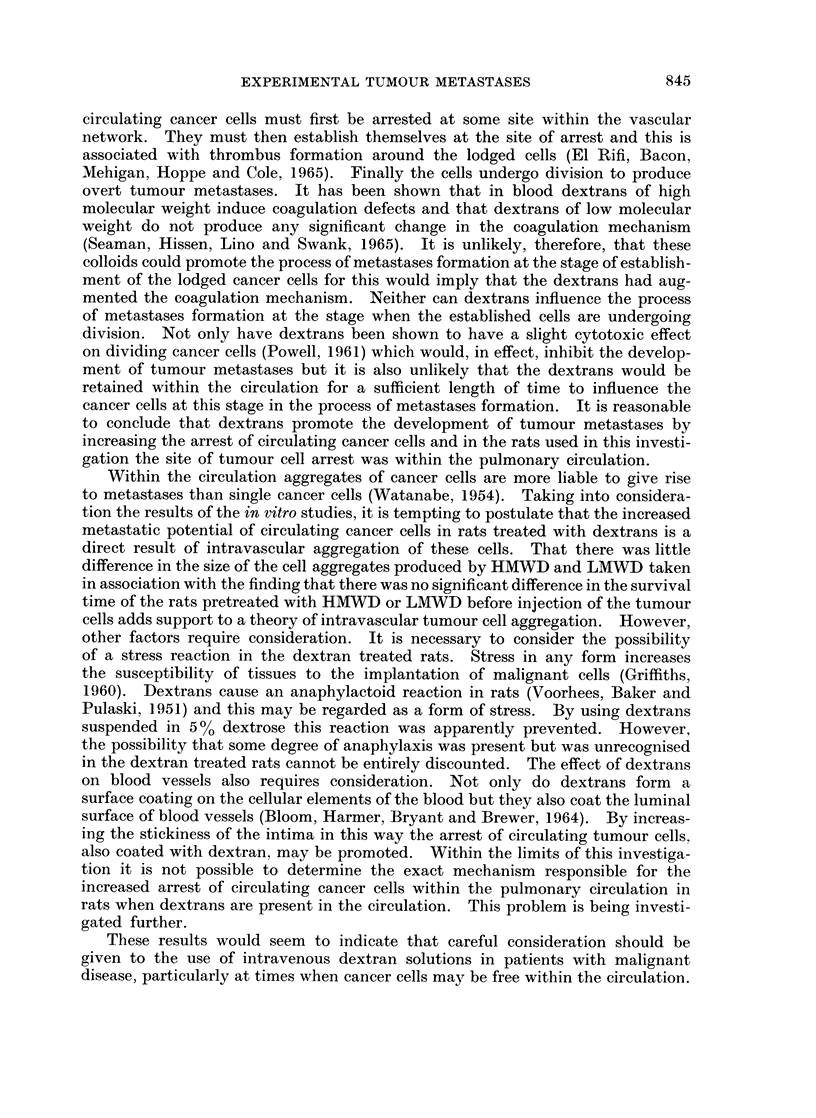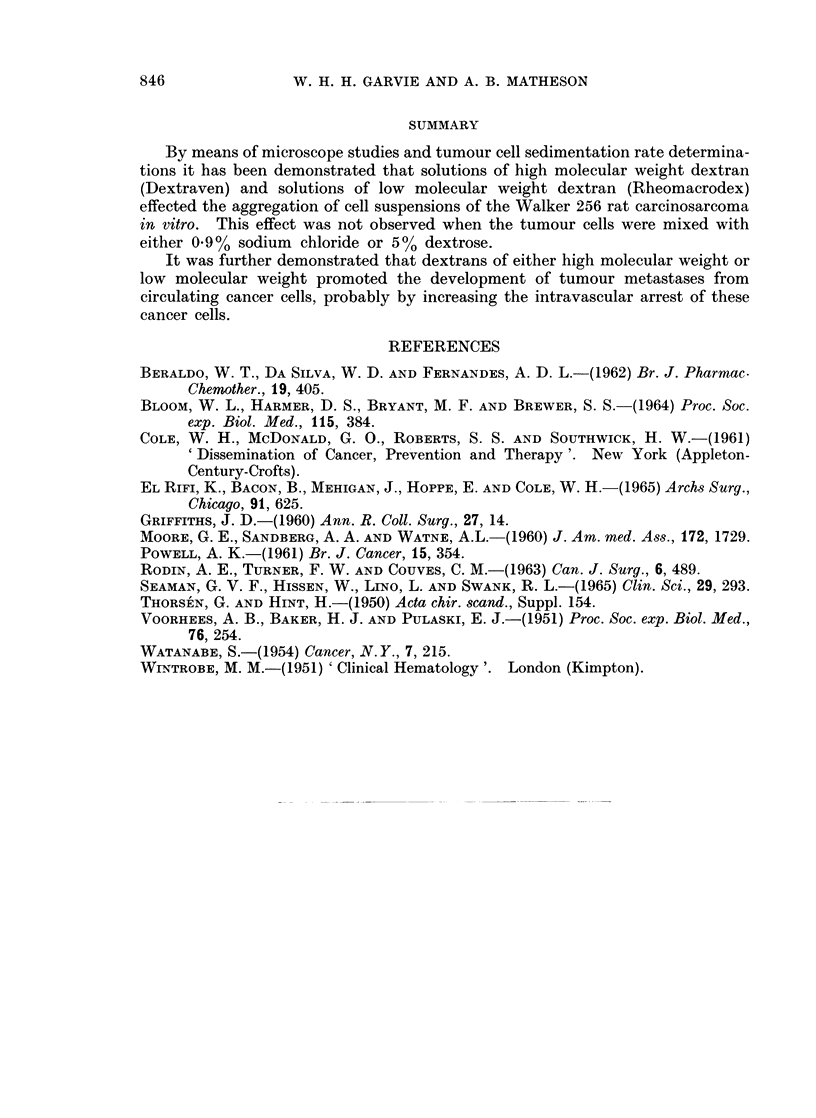# The effect of intravenous fluids on the development on experimental tumour metastases: their effect on tumour cell aggregation.

**DOI:** 10.1038/bjc.1966.96

**Published:** 1966-12

**Authors:** W. H. Garvie, A. B. Matheson


### THE EFFECT OF INTRAVENOUS FLUIDS ON THE DEVELOPMENT OF EXPERIMENTAL TUMOUR METASTASES: THEIR EFFECT ON TUMOUR CELL AGGREGATION

W. H. H. GARVIE AND A. B. MATHESON

*From the Department of Surgery, the Royal Infirmary, Aberdeen*

Received for publication August 18, 1966

STUDIES carried out on patients with malignant disease have demonstrated that operative manipulation of a malignant tumour releases cancer cells into the circulation, frequently in considerable number (Moore, Sandberg and Watne, 1960; Cole, McDonald, Roberts and Southwick, 1961). It is, therefore, of considerable importance during operation to avoid, as far as is possible, any procedure which will promote the development of tumour metastases from these circulating cancer cells.

Many operations for malignant disease entail extensive and lengthy surgical procedures and it has become common practice for patients submitted to this type of surgery to receive intravenous fluids during the course of the operation. The intravenous infusion apparatus, frequently set up some hours before operation, can be used for blood transfusions should this be required. For the purpose of fluid replacement it may be retained for a few days after operation. When blood is not being used, under different circumstances various fluids may be infused into the patient. Little is known about the effect of intravenous fluids on cancer cells and, in particular, the influence these solutions may have on the metastatic potential of circulating cancer cells.

It is the purpose of this experimental study to determine the effect both *in vitro* and *in vivo* of four clinically available intravenous solutions on cell suspensions of the Walker 256 tumour system. Because it is established that tumour cell aggregates are more liable to give rise to metastases than single circulating cancer cells (Watanabe, 1954) particular attention has been paid to the aggregating effect of the intravenous fluids on the experimental tumour cells.

### METHODS

In the present series of experiments the Walker 256 tumour was used in the form of a suspension of single tumour cells prepared by a modification of the method described by Rodin, Turner and Couves (1963). The tumour cells were suspended in Hank's balanced salt solution (BSS).

Four intravenous solutions were used:

1. Dextraven (Benger Laboratories Ltd.)—a sterile solution of 10% w/v dextran, having an average molecular weight of 150,000, in 5% dextrose solution. It is designated a high molecular weight dextran (HMWD) solution.

2. Rheomacrodex (Pharmacia G.B. Ltd.)—a sterile solution of 10% w/v dextran, having an average molecular weight of 40,000, in 5% dextrose solution. It is designated a low molecular weight dextran (LMWD) solution.

3. A sterile dextrose solution containing 50 g. of dextrose/litre of water (5% dextrose).

4. A sterile salt solution containing 9 g. of sodium chloride/litre of water (0·9% sodium chloride).

Both Dextraven and Rheomacrodex are prepared commercially with the dextran fractions suspended either in 0·9% sodium chloride or in 5% dextrose. However, as dextrans cause an anaphylactoid reaction in rats which can be effectively overcome by dextrose (Beraldo, Da Silva and Fernandes, 1962), the dextrose suspended dextran solutions were used in this investigation.

The investigation was divided into three parts. The *in vitro* studies were carried out at room temperature.

(1) *Microscope studies*

Six Walker 256 tumour cell suspensions containing 25,000 cells/c.mm., 30,000 cells/c.mm., 40,000 cells/c.mm., 65,000 cells/c.mm., 75,000 cells/c.mm. and 90,000 cells/c.mm. were used. At each of these cell concentrations a single drop of the suspension was placed on each of five clean glass microscope slides. One drop of the four intravenous solutions under investigation and one drop of Hank's BSS was then placed in contact with the cell suspension on the glass slides, one solution to a slide. The two drops on each slide were gently mixed together and a coverslip placed over the fluid. With the slides horizontally placed on the microscope stage, microscopic examination of the cells was then undertaken.

(2) *Estimation of tumour cell sedimentation rates*

The six Walker 256 tumour cell suspensions prepared for the microscope study were also used to determine tumour cell sedimentation rates. At each cell concentration a total of 5 ml. of suspension was used. This was divided equally into five clean glass test-tubes, 1 ml. to each tube. To the first test-tube was added 1 ml. of Dextraven, to the second 1 ml. of Rheomacrodex, to the third 1 ml. of 0·9% sodium chloride, to the fourth 1 ml. of 5% dextrose and 1 ml. of Hank's BSS was added to the fifth tube. The tubes were shaken to ensure uniform mixing of the solutions with the cell suspension. The resulting preparations were drawn up to the zero mark of standard Westergren sedimentation tubes of 200 gradations and 2·5 mm. internal diameter. These tubes were then clipped into racks, care being taken to ensure that they were vertically placed. The rate of cell sedimentation was read every thirty minutes for the first two hours and thereafter hourly for a total period of nine hours. Because of the decrease in viability of tumour cells with time observations were not continued beyond the nine hour period.

(3) In vivo *studies*

Female albino Sprague-Dawley rats obtained from the Oxfordshire Laboratory Animal Colonies were used. They weighed between 160–200 g. at the beginning of the experiment. Housed six animal to a cage, they were fed a standard cubed diet and supplied with water *ad libitum*.

Three sterile single tumour cell suspensions containing 60,000 cells/c.mm., 30,000 cells/c.mm. and 20,000 cells/c.mm. were prepared.

In this investigation all injections were given under ether anaesthesia into the saphenous vein found in the inner aspect of the hind limb of the rat using a 20-gauge intravenous needle.

The animals were divided at random into three groups with thirty rats in each group.

In the first group of thirty rats the first rat was given a slow intravenous injection of 1 ml. of Dextraven and this was followed one minute later by an intravenous injection of 60,000 Walker 256 tumour cells in 0·1 ml. of Hank's BSS. The second rat was similarly treated but was given 1 ml. of Rheomacrodex in place of 1 ml. of Dextraven. The third, fourth and fifth rats were treated in an identical manner, the Dextraven being replaced by 1 ml. of 0·9% sodium chloride, 1 ml. of 5% dextrose and 1 ml. of Hank's BSS, respectively. This injection sequence was repeated five more times giving a total of six rats injected with each intravenous solution under investigation and six rats injected with Hank's BSS.

In the second group of thirty rats the injection sequence used in the first group of thirty rats was repeated but in this group a tumour cell suspension containing 30,000 cells in 0·1 ml. of Hank's BSS was used in place of the tumour cell suspension containing 60,000 cells in 0·1 ml. of Hank's BSS.

The third group of thirty rats was treated in an identical manner to the other two groups of rats but in this instance a tumour cell suspension containing 20,000 cells in 0·1 ml. of Hank's BSS was used.

All the rats were retained until death and the number of survival days following the intravenous injections were noted. The animals were then subjected to careful post-mortem examination. Macroscopic appearances were noted and selected tissues were excised for microscopic study.

### RESULTS

### (1) *Microscope studies*

At the higher cell concentrations used in this part of the investigation immediate aggregation of the tumour cells into a coherent cell mass was observed on macroscopic examination of the microscope slides when the cell suspensions were mixed with either of the dextran solutions, Dextraven or Rheomacrodex. On examination of this coherent cell mass under the microscope it was found to be composed of clumps of tumour cells, frequently comprising hundreds of cells, connected by bridges of cells of varying thickness. With time these bridges of cells gradually thinned out and finally broke apart leaving discrete compact aggregates of cells. Pressure on the cover-slip did not cause the cell aggregates to disperse into single cells but moved them as solid cell masses. At the lower cell concentrations a similar sequence of events was observed when the tumour cells were mixed with either Dextraven or Rheomacrodex. However, the cell masses were smaller and the bridges of cells connecting them together were thinner. These cellular bridges rapidly broke apart to leave small isolated clumps of tumour cells. Although it was impossible to accurately assess the number of tumour cells making up the discrete cell masses, overall the cell masses appeared slightly larger in the tumour cell suspensions mixed with the HMWD solution than in the tumour cell suspensions mixed with the LMWD solution.

Aggregation of the tumour cells was not observed when the cells were mixed with 0·9% sodium chloride, 5% dextrose or further mixed with Hank's BSS. The cells spread evenly over the microscope slide. Occasionally a tendency to form clumps was observed but gentle pressure on the cover-slip dispersed the cells in the free state once more indicating that true cell aggregation had not taken place.

(2) *Estimation of tumour cell sedimentation rates*

Within minutes of setting up the Westergren sedimentation apparatus obvious aggregation of the tumour cells to form a coherent cell mass was observed in the sedimentation tubes where the Walker 256 tumour cells had been mixed with either Dextraven or Rheomacrodex. The cells formed a dense column in the

FIG. 1.—The 1 hour sedimentation rates of various concentrations of Walker 256 tumour cells suspended in different solutions.

middle of the tube surrounded by a zone of clear fluid. This effect was more apparent at the higher cell concentrations. Cell aggregation was not observed in the sedimentation tubes where the tumour cells had been mixed with 0·9% sodium chloride, 5% dextrose or Hank's BSS.

The coherent mass of cells in the sedimentation tubes gradually broke down to form discrete cell masses. Only after this had occurred did sedimentation of the cells take place. At the lower tumour cell concentrations there was a more rapid break down of the coherent cell mass into discrete cellular particles than at the higher cell concentrations. This resulted in a progressive decrease in the time of onset of sedimentation as the cell concentration fell from 90,000 cells/c.mm. to 25,000 cells/c.mm. In the three solutions where cell aggregation was not observed, the tumour cells started to sediment as soon as the Westergren tubes were fixed in place.

The one hour cell sedimentation rates recorded when the various concentrations of Walker 256 tumour cells were mixed with the different solutions investigated are shown in Fig. 1. The difference in cell sedimentation rate between those solutions which cause aggregation of the tumour cells and those solutions which do not cause the tumour cells to aggregate is obvious, particularly at the higher cell concentrations. Because of the delay in onset of sedimentation caused by aggregation of the tumour cells, the tumour cell suspensions mixed with either of the dextran solutions had the lower readings after one hour. When the tumour cells were mixed with 0·9% sodium chloride, 5% dextrose or Hank's BSS, between concentrations of 90,000 cells/c.mm. and 40,000 cells/c.mm. the graphs of the one hour sedimentation rates showed a linear relationship. This was not a feature of the graphs of the one hour sedimentation rates of the tumour cells suspended in either of the two dextran solutions. Below concentrations of 40,000 cells/c.mm. the linearity was lost, presumably because at these cell concentrations after one hour the upward packing effect of the cells interfered with free cell sedimentation.

FIG. 2.—The sedimentation rates recorded over 9 hours for three different concentrations of Walker 256 tumour cells suspended in either Dextraven or Rheomacrodex.

The sedimentation rate over a nine hour period for three different concentrations of Walker 256 cells mixed with either Dextraven or Rheomacrodex are shown graphically in Fig. 2. The graphs of the three other cell concentrations suspended in either of the dextran solutions followed a similar pattern and have been omitted from Fig. 2 for the sake of clarity. The delay in onset of sedimentation due to the aggregation of the cells into a coherent cell mass is obvious. It is most marked at a concentration of 90,000 cells/c.mm., is present to a lesser extent at a concentration of 65,000 cells/c.mm. and is least obvious at a cell concentration of 40,000 cells/c.mm. At each cell concentration the onset of sedimentation was longer delayed when the tumour cells had been mixed with the HMWD solution. Once initiated, cell sedimentation proceeded at almost the same rate in both dextran solutions. Consequently the sedimentation curves over the period of observation follow almost parallel lines with the sedimentation rate for the Dextraven suspended cells always less, at any given time, than the sedimentation rate for the tumour cells suspended in Rheomacrodex.

(3) In vivo *studies*

All the rats survived the intravenous injections. During the injections a transient rise in both the respiratory rate and the heart rate was noted but both returned to normal within a few minutes of completing the injections. Obvious histamine shock was not observed in any of the animals.

The mean survival time in days and the standard deviation for each group of six rats is shown in Table I. At each of the three cell concentrations used in this part of the investigation the animals treated with either of the two dextran solutions were the first to die when comparison was made with the death rate of the animals injected with the three other solutions. This difference was not so apparent at a cell concentration of 60,000 cells/c.mm., but was obvious at the other two cell concentrations used. There was no significant difference in survival time between the rats treated with Dextraven and the rats treated with Rheomacrodex.

TABLE I.—*The Mean Survival Times and Standard Deviations in Days of Groups of Six Rats Injected with* 1 *ml. of Various Intravenous Fluids Followed by the Intravenous Injection of Walker* 256 *Tumour Cells.*

| | Number of tumour cells injected intravenously | | |
|---|---|---|---|
| Intravenous fluids : | 60,000 | 30,000 | 20,000 |
| Hank's BSS . | 16·5±4·9 | 20·7±1·0 | 26·5±1·6 |
| Saline 0·9% . | 9·4±1·6 | 21·3±1·9 | 27·0±1·9 |
| Dextrose 5% . | 8·9±1·9 | 21·2±1·9 | 26·0±1·1 |
| Rheomacrodex . | 8·2±0·9 | 15·5±0·9 | 19·1±1·1 |
| Dextraven . . | 7·8±1·0 | 15·2±1·4 | 18·7±0·7 |

In each group of six rats all the animals died within a few days of each other with the exception of the rats injected with 1 ml. of Hank's BSS intravenously followed by 60,000 tumour cells. In this group two animals survived for a considerably longer period of time than the remaining four rats thereby increasing the mean survival figure. With this exception, the rats treated with 0·9% sodium chloride, 5% dextrose or Hank's BSS survived for approximately the same number of days at each of the three different cell concentrations investigated.

All the animals died from extensive pulmonary deposits of the Walker 256 carcinosarcoma. These deposits were so extensive that it was frequently difficult to identify normal lung tissue either macroscopically or microscopically in many of the specimens examined. No apparent difference in the pattern of metastases was found between the rats treated with Dextraven or Rheomacrodex and the rats treated with the other three solutions. Macroscopic and microscopic examination of other organs taken from the rats failed to demonstrate tumour metastases at any other site.

### DISCUSSION

The two dextran solutions used in this investigation, Dextraven and Rheomacrodex, aggregated the experimental Walker 256 tumour cells *in vitro*. This property of tumour cell aggregation was not exhibited by 0·9% sodium chloride,

5% dextrose or by Hank's BSS. The dextrans were suspended in 5% dextrose and the Walker 256 tumour cells were suspended in a small volume of Hank's BSS. As neither of these solutions caused the tumour cells to aggregate, the cell aggregation effected by the two dextran solutions must be attributed directly to the dextran fractions.

It may be assumed that the sequence of events observed macroscopically in the Westergren sedimentation tubes parallels the sequence of events observed on the microscope slides when the experimental tumour cells were mixed with either of the dextran solutions. In the sedimentation tubes the cancer cells initially formed a coherent cell mass consisting of zones of aggregated cells joined by bridges of cells of varying thickness. Only after these bridges had disrupted did cell sedimentation start. According to Thorsén and Hint (1950), in studying any cell suspension where there is a strong aggregating tendency it is always possible to distinguish a distinct phase of aggregation and a distinct phase of sedimentation in the column of cells in a sedimentation tube. From the observations made in the present experiment it is apparent that both HMWD and LMWD exerted a strong aggregating effect on the Walker 256 tumour cells used in this investigation. As it was found that the onset of sedimentation was always longer delayed when the tumour cells were mixed with Dextraven compared with the same tumour cell concentration mixed with Rheomacrodex, HMWD must hold the cellular bridges intact between the zones of aggregated cells for a longer time than LMWD. Therefore, HMWD must exert a more prolonged initial aggregating effect on the experimental tumour cells than LMWD.

The chief factor influencing the rate of sedimentation is the size of the sedimenting particle (Wintrobe, 1951). On microscope examination HMWD appeared to produce cell aggregates composed of a larger number of cells than LMWD, although absolute comparisons were impossible due to the difficulty experienced in counting the number of cancer cells in each aggregate with any degree of accuracy. Study of the cell sedimentation rates over nine hours demonstrated that, while there was a greater delay in onset of sedimentation in the Dextraven suspended tumour cells, once sedimentation had started the hourly rate ran almost parallel to the hourly rate of sedimentation for the tumour cells mixed with Rheomacrodex. The sedimentation rate of the tumour cells in the HMWD solution never exceeded, at any given time, the sedimentation rate for the tumour cells in the LMWD solution, a result to be expected had there been a pronounced difference in the size of the sedimenting aggregates of cancer cells. From these results it can be deduced that, although HMWD exerts a more prolonged initial aggregating effect on the experimental tumour cells as determined by the greater delay in onset of sedimentation when compared with LMWD, once this effect had passed off the cell aggregates finally produced by the two dextran solutions used in this investigation are approximately the same size.

Study of the mean survival time of the rats used in this investigation demonstrated that, at each of the three tumour cell concentrations used, the first rats to die from diffuse pulmonary metastases were the rats injected intravenously with either of the dextran solutions before the intravenous injection of cancer cells. It is apparent, therefore, that under the conditions of this investigation the dextran solutions promoted the development of tumour metastases. To understand how this could be effected it is necessary to consider the steps in the formation of blood-borne tumour metastases. To produce tumour metastases circulating cancer cells must first be arrested at some site within the vascular network. They must then establish themselves at the site of arrest and this is associated with thrombus formation around the lodged cells (El Rifi, Bacon, Mehigan, Hoppe and Cole, 1965). Finally the cells undergo division to produce overt tumour metastases. It has been shown that in blood dextrans of high molecular weight induce coagulation defects and that dextrans of low molecular weight do not produce any significant change in the coagulation mechanism (Seaman, Hissen, Lino and Swank, 1965). It is unlikely, therefore, that these colloids could promote the process of metastases formation at the stage of establishment of the lodged cancer cells for this would imply that the dextrans had augmented the coagulation mechanism. Neither can dextrans influence the process of metastases formation at the stage when the established cells are undergoing division. Not only have dextrans been shown to have a slight cytotoxic effect on dividing cancer cells (Powell, 1961) which would, in effect, inhibit the development of tumour metastases but it is also unlikely that the dextrans would be retained within the circulation for a sufficient length of time to influence the cancer cells at this stage in the process of metastases formation. It is reasonable to conclude that dextrans promote the development of tumour metastases by increasing the arrest of circulating cancer cells and in the rats used in this investigation the site of tumour cell arrest was within the pulmonary circulation.

Within the circulation aggregates of cancer cells are more liable to give rise to metastases than single cancer cells (Watanabe, 1954). Taking into consideration the results of the *in vitro* studies, it is tempting to postulate that the increased metastatic potential of circulating cancer cells in rats treated with dextrans is a direct result of intravascular aggregation of these cells. That there was little difference in the size of the cell aggregates produced by HMWD and LMWD taken in association with the finding that there was no significant difference in the survival time of the rats pretreated with HMWD or LMWD before injection of the tumour cells adds support to a theory of intravascular tumour cell aggregation. However, other factors require consideration. It is necessary to consider the possibility of a stress reaction in the dextran treated rats. Stress in any form increases the susceptibility of tissues to the implantation of malignant cells (Griffiths, 1960). Dextrans cause an anaphylactoid reaction in rats (Voorhees, Baker and Pulaski, 1951) and this may be regarded as a form of stress. By using dextrans suspended in 5% dextrose this reaction was apparently prevented. However, the possibility that some degree of anaphylaxis was present but was unrecognised in the dextran treated rats cannot be entirely discounted. The effect of dextrans on blood vessels also requires consideration. Not only do dextrans form a surface coating on the cellular elements of the blood but they also coat the luminal surface of blood vessels (Bloom, Harmer, Bryant and Brewer, 1964). By increasing the stickiness of the intima in this way the arrest of circulating tumour cells, also coated with dextran, may be promoted. Within the limits of this investigation it is not possible to determine the exact mechanism responsible for the increased arrest of circulating cancer cells within the pulmonary circulation in rats when dextrans are present in the circulation. This problem is being investigated further.

These results would seem to indicate that careful consideration should be given to the use of intravenous dextran solutions in patients with malignant disease, particularly at times when cancer cells may be free within the circulation.

SUMMARY

By means of microscope studies and tumour cell sedimentation rate determinations it has been demonstrated that solutions of high molecular weight dextran (Dextraven) and solutions of low molecular weight dextran (Rheomacrodex) effected the aggregation of cell suspensions of the Walker 256 rat carcinosarcoma *in vitro*. This effect was not observed when the tumour cells were mixed with either 0·9% sodium chloride or 5% dextrose.

It was further demonstrated that dextrans of either high molecular weight or low molecular weight promoted the development of tumour metastases from circulating cancer cells, probably by increasing the intravascular arrest of these cancer cells.

### REFERENCES

BERALDO, W. T., DA SILVA, W. D. AND FERNANDES, A. D. L.—(1962) *Br. J. Pharmac. Chemother.*, **19**, 405.

BLOOM, W. L., HARMER, D. S., BRYANT, M. F. AND BREWER, S. S.—(1964) *Proc. Soc. exp. Biol. Med.*, **115**, 384.

COLE, W. H., McDONALD, G. O., ROBERTS, S. S. AND SOUTHWICK, H. W.—(1961) ‘Dissemination of Cancer, Prevention and Therapy’. New York (Appleton-Century-Crofts).

EL RIFI, K., BACON, B., MEHIGAN, J., HOPPE, E. AND COLE, W. H.—(1965) *Archs Surg., Chicago*, **91**, 625.

GRIFFITHS, J. D.—(1960) *Ann. R. Coll. Surg.*, **27**, 14.

MOORE, G. E., SANDBERG, A. A. AND WATNE, A.L.—(1960) *J. Am. med. Ass.*, **172**, 1729.

POWELL, A. K.—(1961) *Br. J. Cancer*, **15**, 354.

RODIN, A. E., TURNER, F. W. AND COUVES, C. M.—(1963) *Can. J. Surg.*, **6**, 489.

SEAMAN, G. V. F., HISSEN, W., LINO, L. AND SWANK, R. L.—(1965) *Clin. Sci.*, **29**, 293.

THORSÉN, G. AND HINT, H.—(1950) *Acta chir. scand.*, Suppl. 154.

VOORHEES, A. B., BAKER, H. J. AND PULASKI, E. J.—(1951) *Proc. Soc. exp. Biol. Med.*, **76**, 254.

WATANABE, S.—(1954) *Cancer, N.Y.*, **7**, 215.

WINTROBE, M. M.—(1951) ‘Clinical Hematology’. London (Kimpton).